# Inter-rater agreement, sensitivity, and specificity of the prone hip extension test and active straight leg raise test

**DOI:** 10.1186/2045-709X-22-23

**Published:** 2014-06-16

**Authors:** Paul A Bruno, David P Millar, Dale A Goertzen

**Affiliations:** 1 Faculty of Kinesiology and Health Studies, University of Regina, 3737 Wascana Parkway, Regina, Saskatchewan S4S 0A2, Canada; 2 Private Practice, Regina, Saskatchewan, Canada

**Keywords:** Reproducibility of results, Sensitivity and specificity, Low back pain, Rehabilitation

## Abstract

**Background:**

Two clinical tests used to assess for neuromuscular control deficits in low back pain (LBP) patients are the prone hip extension (PHE) test and active straight leg raise (ASLR) test. For these tests, it has been suggested examiners classify patients as “positive” or “negative” based on the presence or absence (respectively) of specific “abnormal” lumbopelvic motion patterns. The inter-rater agreement of such a classification scheme has been reported for the PHE test, but not for the ASLR test. In addition, the sensitivity and specificity of such classification schemes have not been reported for either test. The primary objectives of the current study were to investigate: 1) the inter-rater agreement of the examiner-reported classification schemes for these two tests, and 2) the sensitivity and specificity of the classification schemes.

**Methods:**

Thirty participants with LBP and 40 asymptomatic controls took part in this cross-sectional observational study. Participants performed 3–4 repetitions of each test whilst two examiners classified them as “positive” or “negative” based on the presence or absence (respectively) of specific “abnormal” lumbopelvic motion patterns. The inter-rater agreement (Kappa statistic), sensitivity (LBP patients), and specificity (controls) were calculated for each test.

**Results:**

Both tests demonstrated substantial inter-rater agreement (PHE test: Kappa = 0.76, 95% CI = 0.57-0.95, *p* < 0.001; ASLR test: Kappa = 0.76, 95% CI = 0.57-0.96, *p* < 0.001). For the PHE test, the sensitivity was 0.18-0.27 and the specificity was 0.63-0.78; the odds ratio (OR) of “positive” classifications in the LBP group was 1.25 (95% CI = 0.58-2.72; Examiner 1) and 1.27 (95% CI = 0.52-3.12; Examiner 2). For the ASLR test, the sensitivity was 0.20-0.25 and the specificity was 0.84-0.86; the OR of “positive” classifications in the LBP group was 1.72 (95% CI = 0.75-3.95; Examiner 1) and 1.57 (95% CI = 0.64-3.85; Examiner 2).

**Conclusion:**

Classification schemes for the PHE test and ASLR test based on the presence or absence of specific “abnormal” lumbopelvic motion patterns demonstrated substantial inter-rater agreement. However, additional investigation is required to further comment on the clinical usefulness of the motion patterns demonstrated by LBP patients during these tests as a diagnostic tool or treatment outcome.

## Background

It is well-established that the coordination of muscle activity around the lumbopelvic region is vital to the generation of mechanical spinal stability [1,2]. Models illustrating mechanisms by which altered motor control strategies in this region serve as a potential cause and/or effect of LBP have been described by Panjabi [3,4] and others [5-7]. Dysfunctional neuromuscular control strategies (e.g. muscle activation levels, coordination of muscle contractions) could therefore result in “clinical instability”, which has been defined as the loss of the ability of the spine to maintain its pattern of displacement under physiologic loads resulting in no initial or additional neurological deficit, no major deformity, and no incapacitating pain [4]. People with low back pain (LBP) have been shown to demonstrate a variety of neuromuscular control alterations compared to asymptomatic individuals [8-15]. The neuromuscular control strategies used during specific postures or tasks can be objectively quantified and used to provide estimates of spinal stability [1,16]. However, these methods involve the use of advanced technology and mathematical modeling that make them of limited use in a routine clinical setting. It would therefore be valuable to develop practical clinical tests that demonstrate sufficient reliability and validity in assessing the neuromuscular control strategies of LBP patients to help facilitate treatment targeted at correcting specific neuromuscular control deficits. Two tests that have been suggested as having potential in this regard are the prone hip extension (PHE) test [17] and active straight leg raise (ASLR) test [18,19].

The PHE test was originally developed as a means of evaluating for a specific neuromuscular control deficit in the lumbopelvic region. During the test, the patient lays prone and alternately lifts each leg off the table to a height of ~20 cm whilst an examiner observes and/or palpates the gluteus maximus (GM), hamstring (HAM), and erector spinae (ES) muscles to determine their relative order of activation [20-22]. Since these original descriptions, however, many studies have demonstrated that there is not a consistent order of activation in LBP patients or asymptomatic individuals [8,23-28]. Although there is a general consensus that the GM becomes active after the HAM and ES during the test [8,23-28], there is some evidence that the onset of the GM is significantly delayed in LBP patients [8] and asymptomatic individuals who demonstrate certain lumbar spine motion patterns [24]. However, the clinical importance of these findings has not been established since the impact of a delayed onset of the GM during the PHE test on the mechanical stability of the lumbopelvic region has not been reported.

An alternative use for the PHE test has also been proposed [17], namely that clinicians should instead observe for three specific “abnormal” lumbar spine motion patterns during the test: 1) rotation of the lumbar spine such that the spinous processes appear to move toward the side of hip extension, 2) a lateral shift of the lumbar spine toward the side of hip extension, and 3) extension of the lumbar spine. The inter-rater agreement of classifying LBP patients as “positive” or “negative” based on the presence or absence (respectively) of these motion patterns has been shown to be good [17].

The ASLR test was originally described as a clinical tool to evaluate the ability of the sacroiliac joints to effectively transfer loads between the pelvis and legs in females with pregnancy-related pelvic pain [29,30]. More recently, researchers have also commented on this test’s potential usefulness in the assessment of the neuromuscular control strategies of the lumbopelvic region in the general LBP population [18,19]. The test is similar to the PHE test, with the patient supine (rather than prone) and asked to alternately lift each leg away from the table to a height of ~20 cm [19,31]. It has been suggested that an inability to maintain a neutral alignment of the pelvis during the test indicates the presence of a neuromuscular control deficit [19,31-33]. However, there are no published studies related to the inter-rater agreement of classifying patients as “positive” or “negative” based on their inability or ability (respectively) to maintain a neutral pelvic alignment during the test.

In addition, the sensitivity and specificity of these examiner-reported classification schemes have not been reported for either test.

Therefore, the primary objectives of the current study were to investigate: 1) the inter-rater agreement of the examiner-reported classification schemes for these two tests, and 2) the sensitivity and specificity of the classification schemes.

## Methods

### Study design and reporting

The design and reporting for the current study conform with the Guidelines for Reporting Reliability and Agreement Studies (GRRAS) [34].

### Participants

A convenience sample of 30 participants with LBP and 40 asymptomatic controls were recruited to take part in this cross-sectional observational study. The demographic information for the LBP group and control group is presented in Table 1. LBP participants were recruited from local medical, chiropractic, physiotherapy, and massage therapy clinics. Control participants were recruited from the students, faculty, and staff of the University of Regina. All participants were naïve to the purpose of the study and provided written informed consent. The study was approved by the University of Regina Research Ethics Board.

**Table 1:** Demographic information for the low back pain (LBP) group and control group

		**LBP group**	**Control group**
**Gender (#)**	***Males***	10	20
	***Females***	20	20
**Age (years)**	***Mean (SD)***	27.7 (5.9)	27.7 (6.1)
**Height (cm)**	***Mean (SD)***	171.1 (9.8)	173.3 (10.3)
**Weight (kg)**	***Mean (SD)***	71.0 (16.4)	71.2 (17.7)
**LBP duration (months)**	***Mean (SD)***	21.2 (35.1)	-
	***Median (range)***	6.0 (1–168)	-
**NPRS (0–10)**	***Mean (SD)***	5.0 (1.5)	-
	***Median (range)***	5.0 (2–7)	-
**ODI (0–100)**	***Mean (SD)***	16.7 (8.5)	-
	***Median (range)***	14.0 (6-36)	-

*Abbreviations: NPRS* Numerical Pain Rating Scale, *ODI* Oswestry Disability Index.

In addition to the mean and SD, the median and range are also reported for demographic data that did not demonstrate a normal distribution (LBP duration, NPRS, ODI).

No statistically significant (*p* < 0.05) between-group differences were noted for gender, age, height, or weight.

A priori exclusion criteria for *all* participants included: adults under 20 years of age or over 40 years of age; history of hip joint injury or trauma, lumbar spine surgery, spinal arthritic disorders, central nervous system disorders, or neuromuscular disorders; unable to perform painless active hip ranges of motion; true leg length inequality > 1 cm; and currently pregnant or recently post-partum (<1 year) females. Additional exclusion criteria for the *LBP group* included: history of significant trauma or unexplained weight loss; LBP not confined to an area between the lower ribs and gluteal folds with or without referral into the lower limbs above the knees; presence of radicular signs (e.g. myotomal motor weakness, deep tendon reflex differences) or nerve root tension tests (e.g. straight leg raise test) in the lower limb; current episode of LBP was not present for at least one month and on most days over the previous month; and average LBP over the previous week < 2/10 on a Numerical Pain Rating Scale (NPRS) [35]. An additional criterion for the *control group* was a history of any spinal or lower limb injury that prevented the performance of normal activities for at least one day in the previous three months.

### Examiners

Two of the investigators (DM, DG), both of whom are licensed chiropractors with over 30 years of clinical experience, examined and provided classifications (see Procedure section) for all participants. In order to minimize the bias in the classifications provided during the data collection sessions, the examiners were blinded to the group status (i.e. LBP, control) of each participant. They were also not permitted to confer with each other during the testing procedures and recorded their classifications on separate pieces of paper.

Prior to the initiation of data collection, the examiners underwent a joint training phase. At the first meeting, a consensus was achieved between the two examiners regarding the specific procedure and criteria to be used for each test (see Procedure section paragraphs 4 and 5). Following this, three sessions were conducted during which undergraduate student and faculty volunteers performed the tests whilst the examiners discussed their findings and clarified any discrepancies in classifications. Adequate training has been shown to be more important than the examiners’ collective experience with a testing procedure for observation-based clinical tests [36].

### Procedures

All data collection sessions took place in the same room in the Faculty of Kinesiology and Health Studies’ Neuromechanical Research Centre at the University of Regina. Upon presentation, participants were provided with a study information sheet and asked to complete an intake form and informed consent form. The intake form was used to collect demographic data and confirm their eligibility for the study. The LBP participants were also asked to complete a NPRS [35] related to their average pain over the last week and an Oswestry Disability Index [37,38].

**Figure 1:**
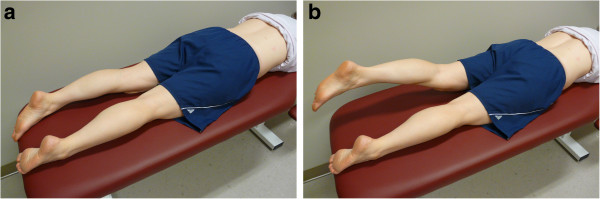
**The start (a) and end (b) positions for the prone hip extension (PHE) test.** The participant is prone with both legs in contact with the bench in the start position **(a)**. The participant’s left leg has been raised off the bench in the end position **(b)**.

**Figure 2:**
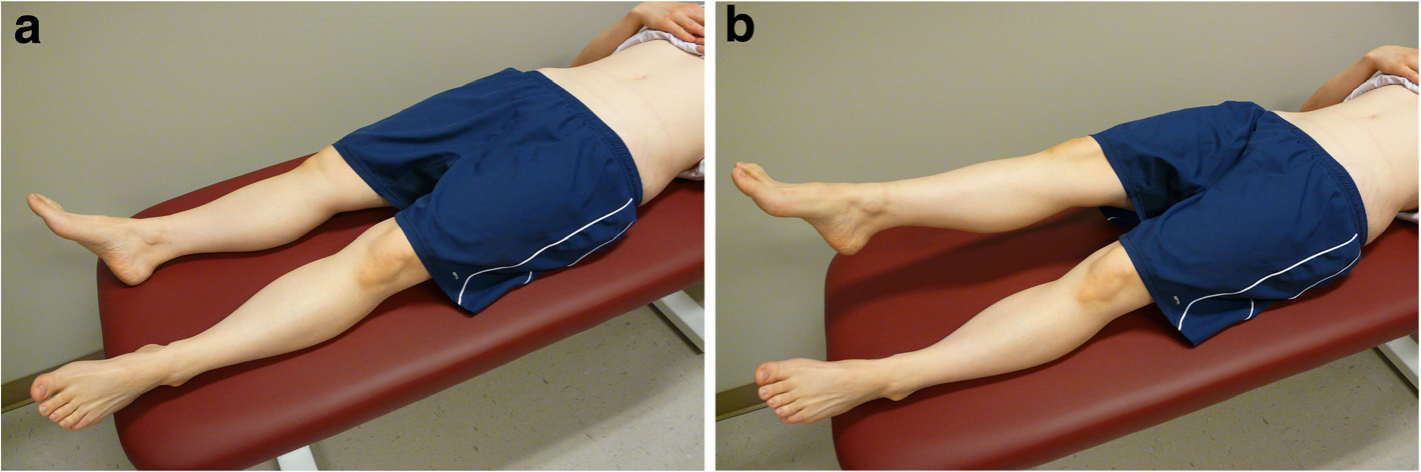
**The start (a) and end (b) positions for the active straight leg raise (ASLR) test.** The participant is supine with both legs in contact with the bench in the start position **(a)**. The participant’s right leg has been raised off the bench in the end position **(b)**.

Participants were required to wear a pair of shorts and lay on a treatment bench. Using a standardized protocol and participant positioning, one of the investigators (PB) instructed the participants on the performance of the two testing procedures. For the PHE test, the participants lay prone and were instructed to alternately lift each leg to a height of ~20 cm and return it to the bench after a 1–2 second hold in the elevated position (Figure 1) [17]. For the ASLR test, the participants lay supine and were instructed to alternately lift each leg to a height of ~20 cm and return it to the bench after a 1–2 second hold in the elevated position (Figure 2) [19,31]. Once the participants were sufficiently familiar with each test, they were allowed to rest for ~ 1 minute before the examiners entered the room.

The participants then performed 3–5 repetitions of each test (performance of the test on both the left and right sides constituted one repetition) whilst the examiners simultaneously observed the performances. The order of test (PHE/ASLR) and leg lifted first (left/right) were randomized to control for order effects and possible fatigue over time. Between each test, the examiners were asked to leave the room and the participants were allowed to rest for ~1 minute.

For the PHE test, the examiners classified each participant as “positive” if one of the following motion patterns was observed during the test: 1) rotation of the lumbar spine such that the spinous processes appear to move toward the side of hip extension, 2) a lateral shift of the lumbar spine toward the side of hip extension, 3) extension of the lumbar spine, or 4) the pelvic girdle raises on the side of hip extension [17]. If none of these motion patterns was observed, the participant was classified as “negative”. The examiners recorded a classification for the participant’s left leg and a classification for the right leg.

For the ASLR test, the examiners classified each participant as “positive” if the pelvic girdle failed to maintain neutral alignment during the test [31-33]. If the pelvic girdle maintained a neutral alignment, the participant was classified as “negative”. The examiners recorded a classification for the participant’s left leg and a classification for the right leg.

### Statistical analyses

For both tests, 2×2 contingency tables were constructed with the classifications provided by Examiner 1 forming the columns and those provided by Examiner 2 forming the rows. The inter-rater agreement for each test was calculated using the kappa statistic and prevalence-adjusted bias-adjusted kappa (PABAK) statistic [39].

For each examiner’s classifications, the sensitivity for both tests was calculated as the “true positive” rate in the LBP group (TP/TP + FN) [40]. The specificity was calculated as the “true negative” rate in the control group (TN/TN + FP) [40]. In addition, the odds ratio (OR) of a “positive” classification (outcome) in the LBP group (exposure) was calculated for both tests.

All statistical analyses were performed using PASW Statistics 18.0 (SPSS Inc, Chicago, IL, USA) and GraphPad InStat 3.10 (GraphPad Software Inc, San Diego, CA, USA) software.

## Results

### Examiner classifications – LBP group

For the PHE test, Examiner 1 classified 16/60 legs (26.7%) as “positive”, and Examiner 2 classified 11/60 legs (18.3%) as “positive” (Table 2). For the ASLR test, Examiner 1 classified 15/60 legs (25.0%) as “positive”, and Examiner 2 classified 12/60 legs (20.0%) as “positive” (Table 3).

**Table 2:** Examiner-reported classifications for the prone hip extension (PHE) test for the low back pain (LBP) group

		**Examiner 1**		
		**Positive**	**Negative**	
**Examiner 2**	**Positive**	11	0	*11*
	**Negative**	5	44	*49*
		*16*	*44*	

**Table 3:** Examiner-reported classifications for the active straight leg raise (ASLR) test for the low back pain (LBP) group

		**Examiner 1**		
		**Positive**	**Negative**	
**Examiner 2**	**Positive**	11	1	*12*
	**Negative**	4	44	*48*
		*15*	*45*	

### Examiner classifications – control group

For the PHE test, Examiner 1 classified 18/80 legs (22.5%) as “positive”, and Examiner 2 classified 12/80 legs (15.0%) as “positive” (Table 4). For the ASLR test, Examiner 1 classified 13/80 legs (16.3%) as “positive”, and Examiner 2 classified 11/80 (13.8%) legs as “positive” (Table 5).

**Table 4:** Examiner-reported classifications for the prone hip extension (PHE) test for the control group

		**Examiner 1**		
		**Positive**	**Negative**	
**Examiner 2**	**Positive**	11	1	*12*
	**Negative**	7	61	*68*
		*18*	*62*	

**Table 5:** Examiner-reported classifications for the active straight leg raise (ASLR) test for the control group

		**Examiner 1**		
		**Positive**	**Negative**	
**Examiner 2**	**Positive**	10	1	*11*
	**Negative**	3	66	*69*
		*13*	*67*	

### Inter-rater agreement (LBP group)

For each test, there was 91.7% overall agreement between the examiners for the classification of legs as “positive” or “negative” (Table 6). Both tests demonstrated substantial inter-rater agreement (Kappa = 0.61-0.80), with lower limits (95% CI) that extend into the range of what is considered moderate agreement (Kappa = 0.41-0.60) [41].

**Table 6:** Inter-rater agreement of the prone hip extension (PHE) test and active straight leg raise (ASLR) test for the low back pain (LBP) group

	**PHE test**	**ASLR test**
**Overall agreement (%)**	**91.7%**	**91.7%**
**Kappa statistic**	**0.76**	**0.76**
*95% CI*	*0.57-0.95*	*0.57-0.96*
*p value*	*p < 0.001*	*p < 0.001*
**PABAK statistic**	**0.83**	**0.83**
*Prevalence index*	0.55	0.55
*Bias index*	0.08	0.05

*Abbreviation: PABAK *Prevalence-Adjusted Bias-Adjusted Kappa.

### Sensitivity, specificity, and frequency of “positive” classifications

Both tests demonstrated relatively poor sensitivity and relatively high specificity (Table 7). The frequency of “positive” classifications was not significantly greater in the LBP group compared to the control group for either test (Table 7).

**Table 7:** Sensitivity, specificity, and the odds ratio (OR) of “positive” classifications in the low back pain (LBP) group for the prone hip extension (PHE) test and active straight leg raise (ASLR) test

	**PHE test**		**ASLR test**	
	***Examiner 1***	***Examiner 2***	***Examiner 1***	***Examiner 2***
**Sensitivity**	**0.27**	**0.18**	**0.25**	**0.20**
*95% CI*	*0.16-0.40*	*0.10-0.30*	*0.15-0.38*	*0.11-0.32*
**Specificity**	**0.78**	**0.85**	**0.84**	**0.86**
*95% CI*	*0.67-0.86*	*0.75-0.92*	*0.74-0.91*	*0.77-0.93*
**OR**	**1.25**	**1.27**	**1.72**	**1.57**
*95% CI*	*0.58-2.72*	*0.52-3.12*	*0.75-3.95*	*0.64-3.85*

## Discussion

The results of the current study suggest that the classification schemes proposed for the PHE test [17] and ASLR test [31-33] demonstrate substantial inter-rater agreement [41], with calculated Kappa values of 0.76 for each test (Table 6). These findings generally agree with those reported by Murphy and colleagues for the PHE test [17]. In the current study, the prevalence of the “positive” test findings for both tests need to be considered when interpreting these values since the kappa statistic is influenced by the relative proportion of “positive” and “negative” test findings [39]. This effect is quantified as a “prevalence index”, which is calculated as the absolute value of the difference between the number of “positive” and “negative” test findings as a proportion of the total number of paired ratings. A very high or very low number of “positive” test findings will result in a “high” prevalence index, which will cause the resulting kappa statistic to be reduced (an effect that is greater for larger kappa values) [39]. The kappa statistic can be adjusted in cases of a high prevalence index by calculating the PABAK statistic [39]. In the current study, the calculated prevalence index for both tests was moderate due to the relatively low number of “positive” test findings in the LBP group. The calculated PABAK statistic values were marginally higher, and moved the reliability of both tests into the “almost perfect” range (Table 6) [41].

The frequency of “positive” test findings was not significantly greater in the LBP group compared to the control group for either test (Table 7). However, there was a non-significant trend for the LBP participants to test “positive” more frequently than the control participants, particularly for the ASLR test. However, it should also be highlighted that the 95% CIs of the calculated ORs were relatively large. The specificity of both tests was relatively high, whilst the accompanying sensitivity values were relatively poor (Table 7). These results suggest that there was a relatively low “false positive” rate in the control group and a relatively high “false negative” rate in the LBP group. The low sensitivity values would seem to question whether observing for the “abnormal” motion patterns used in the current study are an effective tool in assessing the neuromuscular control strategies of the lumbopelvic region in LBP patients. However, the sensitivity values may also reflect the non-specific nature of the diagnostic criteria used for our LBP group. Beyond establishing exclusion criteria to rule out a sinister cause of a participant’s LBP (e.g. tumour, infection) and potential neurological involvement, we did not attempt to localize the source of the participants’ symptoms. Murphy and colleagues [42] have suggested that these two tests may be useful in distinguishing patients with LBP originating in the lumbar spine (PHE test) and the sacroiliac joints (ASLR test). In their study, the participants were divided into sub-groups who met specific criteria to establish the origin of their pain as being either in the lumbar spine or sacroiliac joints. The results indicated that the proportion of “positive” PHE test findings was higher in patients deemed to have pain originating in the lumbar spine, while the proportion of “positive” ASLR test findings was higher in patients deemed to have pain originating in the sacroiliac joints.

It is also possible that the criteria used in the current study to indicate a “positive” test were too general. There may be a sub-group of LBP patients who possess specific neuromuscular control deficits that account for the non-significant increase in “positive” test findings in the current study. The selection of the specific motion patterns used in the current study as being representative of neuromuscular control deficits in the lumbopelvic region during the PHE test [17] and ASLR test [19,31-33] have been based on the clinical observation of LBP patients; however, the clinical importance of an individual’s ability or inability to maintain a neutral alignment of the lumbar spine (PHE test) or pelvic girdle (ASLR test) during these tests has not been established. Patients with a clinical diagnosis of sacroiliac joint pain have been shown to demonstrate quantifiable differences in pelvic motion during standing hip flexion compared to asymptomatic individuals [32]. However, it is unknown whether similar (or other) motion pattern differences exist during the ASLR test. In fact, whether LBP patients demonstrate objective quantifiable differences in lumbar spine or pelvic motion during the PHE test or ASLR test has not been reported. Objectively quantifying the lumbopelvic motion patterns used by LBP patients during these tests may elicit specific motion patterns that are better able to distinguish patients with specific neuromuscular control deficits.

The current study has several additional limitations. First, our sample size was relatively small and confined to one geographical location (Regina, Saskatchewan, Canada). In addition, all of our participants were relatively young adults (20–40 years), and our LBP group did not include individuals with co-morbidities (e.g. LBP with radicular involvement, osteoarthritis, diabetes, heart disease). The generalizability of our results to other populations is therefore questionable. Second, neither of our examiners routinely used the PHE test or ASLR test in clinical practice prior to their involvement in the current study. Although it has been reported that adequate training appears to be more important than the examiners’ collective experience with a testing procedure for observation-based clinical tests [36], these findings only relate to a test involving the knee. Therefore, the examiners’ relative lack of experience with the two tests prior to undergoing the training sessions for the current study may have had an effect on our results. Third, we used a dichotomous scale (“positive” and “negative”) to classify the PHE test and ASLR test findings. The examiners in the current study commented that it may have been preferable to use a graded scale (e.g. 3-point scale, 5-point scale) to rate the participants’ performance during the tests. The potential value of such non-dichotomous scales has not been investigated for these tests. Fourth, since the examiners performed the two tests in relatively quick succession on each participant, recollection bias may have potentially influenced the results. Analysis of the raw data demonstrated that: 1) when the first test was classified as “positive”, the second test was also classified as “positive” 54% of the time (Examiner 1) and 56% of the time (Examiner 2), and 2) when the second test was classified as “positive”, the first test had also been classified as “positive” 44% of the time (Examiner 1) and 45% of the time (Examiner 2). Therefore, the influence of recollection bias on the examiners’ classifications for the second test would appear to have been minimal. Finally, the clinical significance of motion pattern alterations during the PHE test and ASLR test has not been fully established. It has been suggested that neuromuscular control deficits present during these tests may have functional implications for the stability of the lumbopelvic region during static postures and dynamic activities [20-22,29,30]. However, since there are no published studies that have assessed the association between the neuromuscular control strategies used during these tests and activities such as gait, the functional implications of neuromuscular control deficits during the tests are currently unknown [15,43].

## Conclusions

Specific classification schemes for the PHE test and ASLR test based on the presence or absence of certain “abnormal” lumbopelvic motion patterns demonstrate substantial inter-rater agreement. Although the specificity of these schemes also appears to be relatively high, their sensitivity was found to be relatively poor. This may be a reflection of the non-specific nature of the diagnostic criteria used in the current study and/or the presence of a certain sub-group of LBP patients who possess specific neuromuscular control deficits that are detectable using these tests. Additional investigation is required to further comment on the potential clinical usefulness of the motion patterns demonstrated by LBP patients during these tests as either a diagnostic tool or treatment outcome.

## Abbreviations

ASLR: Active straight leg raise; FN: False negative; FP: False positive; LBP: Low back pain; NPRS: Numerical pain rating scale; OR: Odds ratio; ODI: Oswestry disability index; PABAK: Prevalence-adjusted bias-adjusted kappa; PHE: Prone hip extension; TN: True negative; TP: True positive.

## Competing interests

The authors declare they have no competing interests.

## Authors’ contributions

PB conceived and designed the study, and drafted the manuscript. All authors were involved in collecting and analyzing the data, as well as reading and approving the final manuscript.
